# Effectiveness of different behavioral interventions on gestational weight gain, post-partum weight retention and anthropometric measures in pregnancy: A randomized controlled trial

**DOI:** 10.34172/hpp.2022.37

**Published:** 2022-12-10

**Authors:** Vandana Rani, Shabnam Joshi

**Affiliations:** Department of Physiotherapy, Guru Jambheshwar University of Science and Technology, Hisar-125001, Haryana, India

**Keywords:** Exercise, Text messaging, Gestational weight gain, Postpartum weight retention

## Abstract

**Background:** The antenatal and postnatal periods are critical stages in a woman’s reproductive life. Many physical changes occur during pregnancy, such as water retention and excessive weight gain. The aim of the present study is to find out the effectiveness of various behavioral interventions during pregnancy to prevent excessive gestational weight gain (GWG) and postpartum weight retention (PPWR).

**Methods:** In this parallel-group randomized controlled trial, 150 pregnant women with singleton pregnancy, aged 20-30 years, body mass index (BMI)≥18.5 kg/m2 and gestational age of less than 16 weeks were randomly allocated into five groups (N=30 in each group): Group A: Control; Group B: Supervised exercise; Group C: Pedometer; Group D: Text message; and Group E: Pedometer plus text message group. Group B received four supervised exercise sessions per month up to delivery; Groups C and E were urged to increase their levels of physical activity, focusing on pedometer-measured step counts of at least 5000–7500 steps per day on seven consecutive days each month. Group E along with group D also received standard SMS messages about physical activity, diet, motivation, and educational-specific topics.

**Results:** The between-group comparisons revealed a statistically significant reduction in PPWR but insignificant difference in GWG. The greatest reduction in PPWR was found in the supervised exercise group (MD=3.25 kg, 95% CI: [1.75, 4.75], *P*=0.0001 with effect size (η^2^ )=0.155).

**Conclusion:** The study found that the supervised exercise can be seen as an effective way of improving the physical activity level and reducing excessive PPWR in pregnant women.

## Introduction

 The antenatal and postnatal periods are critical stages in a woman’s reproductive life. Many physical changes occur during pregnancy, such as water retention and excessive weight gain and physiological weight gain during pregnancy is crucial for maintaining the fetus’s regular growth and health.^1^ However, excessive weight is a notable public health issue, and 57% of pregnant females attain greater weight as per the recommendation by the Institute of Medicine (IOM).^2^

 In 2009, the IOM issued recommendations for weight gain in pregnancy period based on the pre-pregnancy BMI value. As per the recommendations, pregnant female with pre-pregnancy body mass index (BMI) < 18 kg/m^2^ (i.e. underweight) should attain 12.5-18.0 kg, those with a BMI of 18.5-22.9 kg/m^2^ (i.e. normal BMI) should attain 11.5-16 kg, those with a BMI of 23-24.918 kg/m^2^(i.e. overweight) should attain 7.0-11.5 kg, and those with a BMI > 25 kg/m^2^ (i.e. obese) should attain 5.0-9.0 kg.^3^ The impact of gestational weight gain (GWG) on maternal health outcomes has been identified as a possible risk factor that can affect the well-being of both mother and infant, and this has been directly linked with events occurring during pregnancy.^4^ The total weight gain during pregnancy is determined by numerous factors, of which the most modifiable are physical activities, exercises and dietary intake.^5^

 Excessive GWG is the most compliant risk factor during pregnancy, and both maternal and infant health benefit from its control. In addition, excessive GWG is a major contributing factor for weight retention during post-partum period and for long-term obesity.^6^ It is concurrent with negative short and long-term health outcomes for both mother and child, such as pre-eclampsia, gestational diabetes, stillbirth, congenital malformation and fetal macrosomia.^6-8^

 A key contributing factor for weight retention and obesity after childbirth is physical inactivity.^9^ Indeed, American College of Obstetrics and Gynecology recommends engaging in around 150 min/wk of moderate-intensity physical activity both during and after pregnancy.^10^ It has also been reported that an increase in physical activity from pre-pregnancy to post-partum is associated with better maternal well-being in pregnant females.^11^ Many pregnant women regularly visit healthcare professionals, and are highly motivated for maintaining a healthy lifestyle for effective weight management during pregnancy.^12^

 Various studies have assessed the impact of lifestyle interventions such as physical activity, measured by pedometer, supervised exercises and dietary modification during pregnancy; the results indicate these lifestyle interventions are effective at preventing excessive GWG and reducing post-partum weight retention (PPWR) in normal BMI,^13,14^ overweight and obese pregnant women.^15-17^ In addition, e-health and text message-based interventions are also effective measures for reducing GWG during pregnancy.^18,19^ During pregnancy, women can be motivated to induce behavioral changes; as such, interventions which can regulate the behavior in pregnancy can be helpful in encouraging a healthy lifestyle in the long term.

 A recent study demonstrated that antenatal lifestyle interventions based on physical activity and planned diet were related with a decreased risk of unfavorable maternal and newborn outcomes as well as a decreased risk of GWG.^20^ Although earlier studies have examined the use of exercise,^21^ text message,^18^ physical activity using a pedometer and physical activity plus diet^22^ as separate specific lifestyle interventions, none have assessed the effect of their combined application. Therefore, the present study aims at determining the effectiveness of various behavioral interventions, *viz.* supervised exercises, pedometer, text message and pedometer plus text message, during pregnancy to prevent excessive GWG and PPWR.

## Materials and Methods

###  Study design

 The current study was performed as a parallel-group, five-arm randomized controlled trial. The Institutional Ethical Committee provided ethical approval via letter no. PTY/2018/710A. The study was performed in congruence with the Helsinki Declaration’s ethical standards for human participants in 2013. On January 3, 2019, the current trial was prospectively registered in the Trial Registry of India under CTRI No. CTRI/2019/01/016888.

###  Participants and setting

 The participants consisted of pregnant females recruited from the OPD maternity hospital empanelled with Guru Jambheshwar University of Science and Technology, Hisar, Haryana, India, from March, 2019 to August, 2020. The following inclusion criteria were applied during recruitment: singleton pregnancy, aged 20-30 years, gestational age of less than 16 weeks at inclusion, BMI ≥ 18.5 kg/m^2^, ability to talk and read Hindi and English languages and access to a mobile phone. The exclusion criteria comprised age over 30 years, twin or multiple gestations, BMI > 30 kg/m^2^, high-risk pregnancy, declared unfit to perform exercises by health professionals, any pre- pregnancy complications such as hypertension, diabetes and any significant health conditions limiting participation in physical activity.

 All of the pregnant women who were included in the study had similar socioeconomic backgrounds and were sedentary prior to enrollment. A total of one hundred fifty women were recruited in the study, and were examined for a duration of eight months, i.e. during the second and third trimester, plus a two-month postpartum period. Before the commencement of the trial, all study participants gave their informed consent.

###  Recruitment and interventions

 As, due to various cultural and social beliefs, women do not typically engage in physical activity or exercise during pregnancy, recruitment of pregnant females was difficult. To combat this, all pregnant females attending the gynecologist for routine check-ups at the hospitals were given the relevant posters and pamphlets outlining the usefulness of physical activity and exercise during pregnancy. Any willing participants were approached for participation in the study, and screened for participation following the inclusion and exclusion criteria given above.

 The selected participants were then randomly allocated into five groups based on a computer-generated random number table. Block randomization was used for allocating the participants into respective groups. After randomization, the participants were given interventions as per the respective groups. In addition to the interventions suggested by the present study, the pregnant women in each group were given conventional prenatal care from the gynecologist. The basic demographic details, such as height, weight, gestational age, parity, pre-pregnancy weight and BMI were taken at the baseline visit. The outcome variables were measured at recruitment (weeks 14-16), delivery (weeks 36-38) and two months post-partum.

 Control group (group A): The participants in the control group were given advice on how to lead a healthy lifestyle during the baseline visit, with a focus on eating well and exercising while pregnant. They were not discouraged from engaging in their own exercise, however.

 Supervised exercise group (group B): The participants attended antenatal exercise sessions once a week from weeks 15 to 36 of gestation by the principal investigator of the study. The intensity of exercises was kept light to moderate and was customized to the physical state of the participant. The supervised exercise session consisted of 45-60 minutes of primary stretching of all muscle groups, relaxation, breathing exercises, abdominal exercises, strengthening exercises, back care exercises and Kegel exercises for pelvic floor strengthening; these were assigned to the participants based on gestational age and individual ability (Supplementary file 1). The participants were asked to repeat the similar exercises at home at least three days per week and were also motivated to walk for at least 30 minutes for four days per week, until delivery. To monitor the pace of walking and exercises, Borg scale of perceived exertion and the talk test were used. All participants were instructed on when to stop exercising according to the American College of Obstetrics and Gynecology’s exercise guidelines.

 Pedometer group (group C):The women in the pedometer group were encouraged to walk with a goal of at least 5000-7500 steps per day (i.e. low-active category of physical activity) as per recommendations for assessing the physical activity level.^23^ The step counts, as an indicator of physical activity, were measured by an Omron HJ-320 Tri-Axis Pedometer. The participants were instructed regarding the use of the pedometer: they were asked to keep the instrument at their waist level at all times during the day, from the time they get out of bed in the morning until they go to bed at night, except during bathing. They were motivated to do physical activity at moderate intensity with pedometer (12-14 on Borg scale of perceived exertion) without feeling worn out and drained. The females were also instructed to note the daily steps measured by pedometer in a journal, which was reviewed by the investigator on their subsequent visit. The step counts of seven consecutive days of each month were taken and examined.^24^

 The text message group (group D): In this group, the participants were sent text messages that focused on general health and well-being, balanced diet, motivating to improve their physical activity level. Text messages specific to their gestational age and pregnancy myths prevalent in India were also delivered. In total, 42 messages were prepared with the help of gynecologist. These messages were sent to the participants through mobile phone-based text messages, twice weekly and delivered between weeks 15 to 36 of pregnancy (Supplementary file 2).

 Pedometer plus text message group (group E): The pregnant women in this group were given the pedometer to encourage physical activity, with similar instructions as in pedometer group, and text messages similar to the text message group.

###  Outcome measures

 The primary outcome measures were GWG, PPWR, step counts, waist circumference, hip circumference, body mass index and waist-to-hip ratio. The secondary outcome measures comprised infant birth weight and obstetric and neonatal complications. The waist circumference was measured using a non-stretchable measuring tape parallel to the floor midway between iliac crest and lowermost margin of the ribs or at the level of umbilicus. The hip circumference was measured with the subject wearing minimum clothes using a non-stretchable measuring tape parallel to the floor at the maximum circumference of buttocks. The waist-to-hip ratio was calculated as difference between waist circumference and hip circumference. For measuring physical activity, the mean steps count was measured for seven consecutive days each month from month’s four to nine of pregnancy and at two months post-delivery. The details of the assessment of outcome measures are same as described in the current study’s pilot paper.^25^

###  Sample size estimation

 The sample size was determined using Minitab software. Data gathered from the pilot study for PPWR suggested a minimum number of 26 participants per intervention arm at 95% power and 5% significance level. Estimating a dropout rate of 15%, 30 participants per arm were enrolled (total of 150 participants in all five groups).

###  Randomization and allocation concealment

 The participants were selected according to the eligibility criteria and then assigned randomly to four intervention groups and one control group by computer-generated random number table; Group A: Control; Group B: Supervised exercise; Group C: Pedometer; Group D: Text message; and Group E: Pedometer plus text message group. Block randomization was used for allocating the participants into respective groups. The random allocation sequence was produced by the research assistant, who then allocated the participants to the intervention groups. Allocation concealment was done using sealed opaque envelopes and was not revealed to the participants until they were assigned to the respective groups. The participants were enrolled by the investigator. Because of the nature of the research, both the investigator and the participants were unblinded to the interventions. The statistician was blinded to the group allocation for analysis of data (defined as group A to group E).

###  Statistical analysis

 The data were presented as mean and SD, percentage and frequency. Kolmogorov-Smirnov test was used to check the normality of the study. Between-groups comparison was performed using one-way analysis of variance (ANOVA). Post hoc analysis was performed for the significant variables using LSD multiple comparisons. Within-group comparisons were performed using the paired *t* test. Mean difference (MD) along with 95% confidence intervals (CIs) was noted for the significant values. Partial eta squared (η^2^) effect size was calculated and classified into small, medium and large as 0.01, 0.06 and 0.14. An analysis of covariance (ANCOVA) was done to test the GWG and PPWR outcomes between groups, after adjustment for age and BMI as covariates. The principle of intention-to-treat was not used in the study. Completer analysis was used because number of incomplete cases was small (n = 4) and analysis was only performed for complete cases. Observations with missing data were not included in the study. Statistical analyses were conducted using IBM SPSS Statistics 21.0 version. The significance level was set at α = 0.05.

## Results

###  Participant flow 

**Figure 1:**
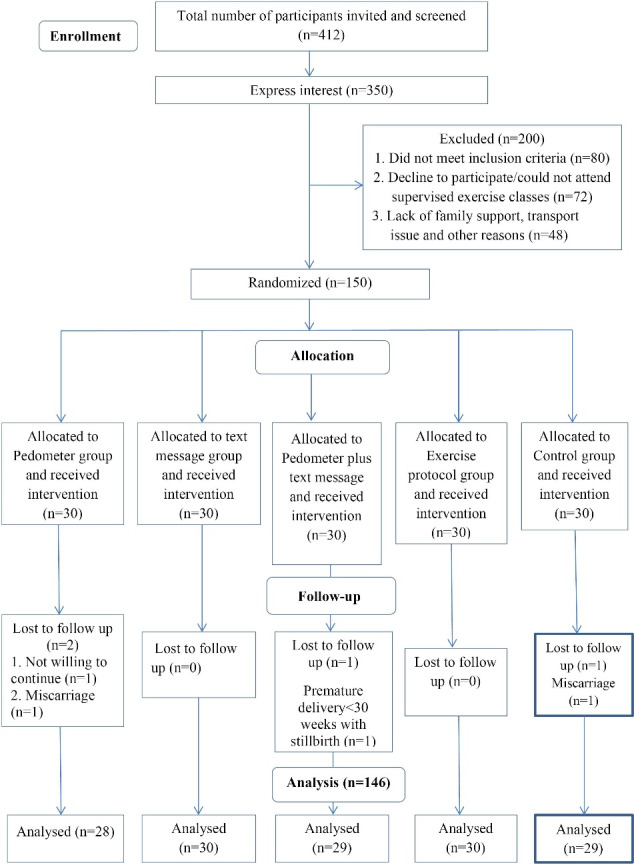
CONSORT flow diagram chart

 In total, 412 pregnant women were screened for inclusion in the study, of whom 350 expressed an interest to participate, i.e. with a response rate of 85%. On screening, 80 participants did not fit the inclusion criteria, 72 participants refused to take part and were not interested in supervised exercise sessions and 48 refused to participate in the study due to the lack of assistance from the spouse and family members, transport issues and other reasons. A total of 150 pregnant females were therefore recruited for the present study and randomised into five groups (n = 30 per group). Of these, 146 pregnant women completed the study. The details of the study are shown in the CONSORT flow diagram given in Figure 1.

###  Recruitment

 The total duration of the study was eight months, including a two-month post-partum follow-up period starting from the date of recruitment in March, 2019 until August, 2020.

###  Baseline data

 The mean age of the participants was 26.12 ± 2.69 years, height was 1.63 ± 0.05m, pre-pregnancy weight was 58.28 ± 7.53 kg, and BMI was 22.40 ± 2.05 kg/m^2^. Out of the screened participants, 71%of women were primiparous. The demographic characteristics were found to be similar at baseline and were statistically insignificant on between group comparisons (Table 1).

**Table 1:** Baseline characteristics of included pregnant women

**Characteristics**	**Control group (n=29)**	**Supervised exercise group (n=30)**	**Pedometer** **group (n=28)**	**Text message** **group (n=30)**	**Pedometer plus text message group (n=29)**	***P*** ** value**
Age		26.14 ± 2.80	26.66 ± 2.22	25.53 ± 2.67	25.77 ± 3.06	26.57 ± 2.64	0.429
Height (m)		1.63 ± 0.05	1.63 ± 0.06	1.64 ± 0.04	1.64 ± 0.05	1.61 ± 0.06	0.235
Baseline weight (kg)		59.34 ± 6.96	57.09 ± 7.11	58.66 ± 7.56	58.97 ± 9.49	57.30 ± 6.32	0.722
Baseline BMI (kg/m^2^)		22.80 ± 2.62	21.92 ± 2.46	22.52 ± 2.83	22.37 ± 2.96	22.42 ± 2.43	0.798
Baseline WC (cm)		81.97 ± 7.45	79.97 ± 7.15	82.17 ± 6.95	80.83 ± 7.14	79.79 ± 6.64	0.591
Baseline HC (cm)		92.83 ± 7.45	89.83 ± 6.60	91.03 ± 7.11	92.70 ± 7.61	90.29 ± 6.85	0.369
Baseline W/H ratio		0.89 ± 0.02	0.87 ± 0.03	0.88 ± 0.02	0.89 ± 0.03	0.88 ± 0.03	0.072
Primiparous (N %)		20/29(68.96)	24/29(82.75)	19/30(63.33)	22/30(73.33)	19/28(67.86)	0.543
Blood pressure	SBP	119.31 ± 6.37	119.83 ± 10.04	115.33 ± 9.37	121.87 ± 8.97	117.50 ± 6.60	0.061
	DBP	79.03 ± 8.51	70.87 ± 7.64	75.00 ± 7.79	76.10 ± 9.11	76.07 ± 9.36	0.080
Blood sugar		102.50 ± 10.70	100.38 ± 8.00	104.38 ± 10.02	101.33 ± 9.94	99.79 ± 9.76	0.471
Job (Yes/no), N		10/19	12/17	10/20	8/22	7/21	0.682
Urban/Rural, N		16/13	16/13	19/11	10/20	19/9	0.078
Education, N (%)	10^th^	1 (3.40)	0 (0.00)	0 (0.00)	0 (0.00)	0 (0.00)	0.287
	12^th^	2 (6.90)	0(0.00)	3 (10.00)	5 (16.70)	3 (10.70)	
	UG	17 (58.60)	14 (48.30)	15 (50.00)	11 (36.70)	10 (35.70)	
	PG	9 (31.00)	14 (48.30)	12 (40.00)	14 (46.60)	15 (56.60)	
	PhD	0 (0.00)	1 (3.40)	0 (0.00)	0 (0.00)	0 (0.00)	

Abbreviations: BMI, body mass index; WC,*waist circumference*, HC, hip circumference; W/H, waist/hip; SBP, systolic blood pressure; DBP, diastolic blood pressure. Data are expressed as mean ± standard deviation (SD) values for all included participants.

###  Outcomes and estimation

 The between-group comparisons revealed a statistically significant reduction in PPWR but insignificant difference in GWG; in addition, a statistically significant difference in birth weight was also observed (Table 2). The post hoc multiple comparisons of PPWR revealed significant reductions in three groups as compared to controls: viz. supervised exercise, pedometer plus text message and pedometer. The greatest reduction in PPWR was found in the supervised exercise group (MD = 3.25 kg; 95% CI: 1.75, 4.75; P = 0.0001**) followed by the pedometer plus text message group [MD = 2.81 kg; 95% CI: 1.30, 4.33; P = 0.0001**] and pedometer group (MD = 1.88 kg, 95% CI: 0.39, 3.36, P = 0.014*) as compared to the control group.

**Table 2:** The between group comparisons for GWG, PPWR and birth weight by one-way ANOVA

**Variables**	**Control group (n=29)**	**Supervised exercise group (n=30)**	**Pedometer group (n=28)**	**Text Message** **group (n=30)**	**Pedometer plus text message group (n=29)**	***P*** ** value/ Effect size (η** ^ 2 ^ **)**
GWG (kg)	12.76 ± 3.85	11.12 ± 3.10	11.94 ± 3.56	12.36 ± 2.53	11.38 ± 3.45	0.31/ 0.03
PPWR (kg)	8.62 ± 3.08	5.36 ± 3.23	6.74 ± 2.69	7.83 ± 2.29	5.80 ± 3.10	0.0001**/0.15
Birth weight (kg)	2.70 ± 0.23	2.89 ± 0.22	2.88 ± 0.23	2.83 ± 0.24	2.93 ± 0.32	0.005**

Abbreviations: GWG, Gestational weight gain; PPWR, Post-partum weight retention. Data are expressed as mean ± standard deviation (SD). ** Significant at *P* < 0.001

 Between-subject comparisons revealed a large effect size for postpartum weight retention (η^2^ = 0.155). In addition, pre-post comparisons in all five groups revealed significant improvements in weight, waist circumference, hip circumference and body mass index, but not waist/hip ratio (Table 3).

**Table 3:** Pre-pregnancy and post-partum two months’ anthropometric measures

**Groups**	**Variables**	**Pre-pregnancy**	**Post-partum two months**	**Mean difference**	***P*** ** value**
Control group (n = 29)	Weight (kg)	59.34 ± 6.96	67.97 ± 8.22	8.62	0.0001**
	WC (cm)	81.97 ± 7.45	91.55 ± 5.67	9.59	0.0001**
	HC (cm)	92.83 ± 7.45	101.41 ± 6.15	8.59	0.0001**
	W/H ratio	0.89 ± 0.02	0.91 ± 0.03	0.02	0.13
	BMI (kg/m^2^)	22.80 ± 2.62	26.51 ± 3.08	3.71	0.0001**
Supervised exercise group (n = 30)	Wt. (kg)	57.09 ± 7.11	62.45 ± 6.68	5.36	0.0001**
	WC (cm)	79.97 ± 7.15	84.62 ± 6.43	4.66	0.0001**
	HC (cm)	89.83 ± 6.60	94.21 ± 4.73	4.38	0.0001**
	W/H ratio	0.87 ± 0.03	0.88 ± 0.04	0.01	0.25
	BMI (kg/m^2^)	21.92 ± 2.46	24.09 ± 2.22	2.18	0.0001**
Pedometer group (n = 28)	Wt. (kg)	58.66 ± 7.56	65.40 ± 8.56	6.74	0.0001**
	WC (cm)	82.17 ± 6.95	89.53 ± 6.85	7.37	0.0001**
	HC (cm)	91.03 ± 7.11	97.73 ± 5.13	6.70	0.0001**
	W/H ratio	0.88 ± 0.02	0.90 ± 0.04	0.02	0.05
	BMI (kg/m^2^)	22.52 ± 2.83	25.14 ± 3.38	2.62	0.0001**
Text Message group (n = 30)	Wt. (kg)	58.97 ± 9.49	66.80 ± 9.54	7.83	0.0001**
	WC (cm)	80.83 ± 7.14	88.40 ± 6.51	7.57	0.0001**
	HC (cm)	92.70 ± 7.61	99.83 ± 6.17	7.13	0.0001**
	W/H ratio	0.89 ± 0.03	0.90 ± 0.03	0.01	0.06
	BMI (kg/m^2^)	22.37 ± 2.96	27.03 ± 3.67	3.63	0.0001**
Pedometer plus text message group (n = 29)	Wt. (kg)	57.30 ± 6.32	63.11 ± 5.79	5.80	0.0001**
	WC (cm)	79.79 ± 6.64	85.77 ± 7.22	5.98	0.0001**
	HC (cm)	90.29 ± 6.85	95.14 ± 6.04	4.86	0.0001**
	W/H ratio	0.88 ± 0.03	0.90 ± 0.03	0.02	0.23
	BMI (kg/m^2^)	22.52 ± 2.83	25.14 ± 3.38	2.62	0.0001**

Abbreviations: BMI, body mass index; WC,*waist circumference*, HC, hip circumference; W/H, waist/hip.

###  Physical activity

 From months 4 to 7, the daily step counts estimated by pedometer in group C were similar to group E: the mean step counts were 4574 ± 1652 and 4273 ± 1132 steps/day in month 4, 4903 ± 1422 and 5105 ± 1230 steps/day in month 5, 4954 ± 1398 and 5492 ± 1071 steps/day in the month 6 and 5257 ± 1233 and 5862 ± 1328 steps/day in month 7. From months 8 and 9, a significantly higher step count was seen in group E compared to group C. Further, group C and group E reported 5324 ± 1175 and 6377 ± 1644 steps/day (*P* = 0.008) in month 8, and 5572 ± 1141 and 6379 ± 996 steps/day (*P* = 0.006), respectively, in month 9.

###  Ancillary analyses

Table 4 summarizes the results of ANCOVA for age and BMI on GWG and PPWR. BMI was found to have a significant influence on GWG (*P* = 0.0001**). The interaction between group and BMI was insignificant. There was no significant effect of age on GWG. Only BMI appeared to be a covariate for GWG. For PPWR, both age and BMI did not show any significant effect.

**Table 4:** Effects of covariates on GWG and PPWR by ANCOVA

**Dependent Variable: GWG and PPWR**									
**Source**	**Type III sum of squares**		**DF**		**Mean square**		**F-value**		***P*** ** value**	
	**GWG**	**PPWR**	**GWG**	**PPWR**	**GWG**	**PPWR**	**GWG**	**PPWR**	**GWG**	**PPWR**
Group	22.589	61.441	4	4	5.647	15.360	0.559	1.925	0.693	0.110
BMI	232.313	11.618	1	1	232.313	11.618	22.998	1.456	0.0001**	0.230
Age	22.597	0.086	1	1	22.597	0.086	2.237	0.011	0.137	0.918
Group * BMI	29.277	91.407	4	4	7.319	22.852	0.725	2.864	0.577	0.026*
Group * Age	32.230	20.288	4	4	8.057	5.072	0.798	0.636	0.529	0.638
Error	1323.286	1045.124	131	131	10.101	7.978				
Total	1648.510	1394.218	145	145						

R squared = 0.197 (Adjusted R squared = 0.111) for GWG. R squared = 0.250 (Adjusted R squared = 0.170) for PPWR.

###  Adverse effects

 During the intervention period, no participants have reported any serious injury or adverse effects.

## Discussion

 The present study examined the effect of different behavioral interventions on GWG, PPWR and anthropometric measures during pregnancy and during a two-month post-partum follow up. Our findings indicate a greater reduction in PPWR in the supervised exercise group compared to the other groups. Hence, supervised exercises would seem to be an effective approach for maintaining optimal weight during the post-partum period. Previous studies have also reported that interventions based on physical activities and exercises were effective in reducing PPWR.^26,27^

 Between-group comparisons indicated a statistically insignificant reduction in GWG in all five groups. Although the results were statistically insignificant, the mean weight gain among the participants of the study was 11.91 ± 3.33 kg, which is an optimal weight gain in pregnancy as per WHO guidelines. It shows that the interventions given in the present study have contributed in restricting excessive weight gain during pregnancy. Previous research based on supervised exercises and pedometer-based physical activity interventions suggest that these interventions do help pregnant women in gaining less gestational weight.^21,22,28^ The pregnant women in group B and group E had, on average, 1.38 to 1.64 kg less GWG compared to the control group. Therefore, our present findings on the effect of physical activity on GWG are consistent with those of previous literature.^14,29^

 The GWG in each group showed that 45% of pregnant women in group B gained weight within recommended guidelines, followed by group E (42%), group C (40%), group D (40%) and group A (34%), which is consistent with the guidelines given by IOM for weight gain.^3^ In group B, only 24% of pregnant females attained weight above the recommended levels, followed by group E (32%), group C (37%), group D (40%) and group A (44%).

 Our findings also identified statistically-significant differences in selected anthropometric measures (weight, waist circumference, hip circumference and BMI) between pre- and post-intervention in all five groups except in waist/hip ratio. In addition, significant differences in mean anthropometric measures were observed in group B, i.e. supervised exercise intervention, from month 4 of pregnancy to second month post-partum, followed by group E, group C, group D and group A. This finding was found to be similar with the finding of Pawalia et al.^30^

 Our between-group comparison also indicated significant improvements in new-born birth weight also; a lower number of caesarean sections were reported in the intervention groups. This result was found to be consistent with the findings of the previous studies.^31,32^

 The present study focused on motivating pregnant women and helping them be more physically active during their pregnancy period. As a result, significant improvements in step counts were observed in the last trimester of pregnancy. However, this is not always the case: while some studies have also demonstrated a similar trend of physical activity among pregnant women,^33^ others have reported lower step counts in the last trimester of pregnancy.^34,35^ The possible reason for increase in steps count seen in the study can be the concern of having a normal and complication-free delivery, motivating the participant to be more physically active during the last trimester of pregnancy.

 A key novelty of the current study is the use of supervised exercise sessions together with the use of a pedometer and text messages as a motivating tool to boost the levels of physical activity in the participants. A pedometer is easy to use, inexpensive and hence, an excellent motivating tool to escalate the physical activity levels in pregnant women. Additionally, the present study recruited pregnant women of all BMIs and from both the rural and urban population, as such; our findings demonstrate broad generalizability, strong external validity and wide applicability. They also show a high retention rate (97%), indicating that the participants liked and accepted the interventions to maintain a healthier lifestyle during pregnancy.

###  Strengths and limitations

 The strengths of the current study comprise the implementation of five different interventions from months 4 to 9of pregnancy, and included a two-months post-delivery follow-up. The study had a dropout rate of 2.67%, which shows a high compliance rate. It also used both objective and subjective measures for assessing physical activity. The primary limitations of the present study are that physical activity assessment was self-reported, and hence the validity of data was dependent on the honesty of the participants; in addition, one-way text messaging was used and the post-partum follow up period was relatively short.

###  Recommendations for future studies

 Further studies are needed to evaluate a more long-term post-partum follow up period, and to determine the influence of early counselling to estimate the benefits of antenatal exercises and physical activity before the start of the pregnancy period and during the first 3 months of the pregnancy. These studies could also investigate the effect of two-way text messaging and ways to improve engagement and compliance with the interventions.

###  Practical implications and policymaking

 The finding of the current study imply that light- to moderate-intensity exercise and the application of a pedometer is an effective and feasible tool to control GWG and PPWR in pregnant women, irrespective of BMI. The use of supervised exercise training during pregnancy should be recommended by the gynaecologist and healthcare professionals to promote the benefits of physical activity in improving foetal and maternal outcomes and general well-being in pregnant women.

## Conclusion

 The study found that the supervised exercise can be seen as an effective way of improving the physical activity level and reducing excessive PPWR in pregnant women. The use of supervised exercises and pedometer, combined with text messages, can be used as a potential approach for practicing a healthy lifestyle during pregnancy.

## Acknowledgements

 We would like to acknowledge Dr. Satya Savant, hospital staff and the participants for their effort and significant contribution to the present study.

## Author Contributions

**Conceptualization: **Vandana Rani, Shabnam Joshi.

**Data curation: **Vandana Rani.

**Formal Analysis: **Vandana Rani, a statistican.

**Investigation: **Vandana Rani.

**Methodology: **Vandana Rani, Shabnam Joshi.

**Project administration: **Vandana Rani.

**Resources: **Vandana Rani, Shabnam Joshi.

**Software: **Vandana Rani, a statistican.

**Supervision: **Vandana Rani, Shabnam Joshi.

**Validation: **Vandana Rani, Shabnam Joshi.

**Visualization: **Vandana Rani, Shabnam Joshi.

**Writing – original draft: **Vandana Rani.

**Writing – review & editing: **Vandana Rani, Shabnam Joshi.

## Funding

 None.

## Ethical Approval

 Ethical approval was taken from Institutional Ethical Committee vide letter no. PTY/2018/710A that followed the ethical standards for human participants declared in Helsinki, 2013. All the participants gave their informed consent before the commencement of the trial.

## Competing Interests

 None.

## Supplementary Files


Supplementary file 1 contains a brief description of supervised exercises.

Supplementary file 2 contains various text messages used in Text Message and Pedometer plus Text Message groups.
