# Supplementary material for: Effectiveness of different behavioral interventions on gestational weight gain, post-partum weight retention and anthropometric measures in pregnancy: A randomized controlled trial
Source: Health Promot Perspect. 2022 Dec 10;12(3):286–94. doi: 10.34172/hpp.2022.37 (PMC9808912; doi:10.34172/hpp.2022.37)
Supplement: Supplementary file 1 — contains a brief description of supervised exercises. [file hpp-12-286-s001.pdf]

Rani et al, **Health Promotion Perspectives,** 2022, 12(3), 286-294
doi: 10.34172/hpp.2022.37
https://hpp.tbzmed.ac.ir

Effectiveness of different behavioral interventions on gestational weight gain, post-partum weight retention and anthropometric measures in pregnancy: A randomized controlled trial

Vandana Rani*, Shabnam Joshi

Department of Physiotherapy, Guru Jambheshwar University of Science and Technology, Hisar-125001, Haryana, India

Supplementary file 1. A brief description of supervised exercises.

Activity category	Duration	Exercises
**Relaxation**	5 minutes	"OM" Chanting<br>Focusing and feeling of breath
**Breathing exercises**	5 minutes	Deep breathing<br>Alom vilom
**Warm-up**	5 minutes	On spot walking<br>Slow stretches for neck, arms, and leg muscles<br>Half squats<br>Half lunges<br>Standing side stretch
**Stretching exercises**	10-15 minutes	Neck- flexion, extension, side stretching and rotations<br>Shoulder - clockwise and anticlockwise circumduction<br>Biceps and triceps stretches<br>Pectoral stretch and retraction<br>Tall standing on heels with hand elevated<br>Trunk- standing side flexion and rotation<br>Thigh- Quadriceps and hamstrings stretches<br>Leg- calf stretch<br>Wide legged forward stretch<br>Triangle pose<br>Butterfly stretch<br>Sitting side stretch
**Abdominal and back care**	10-15 minutes	Spinal trunk rotations in crook-lying position<br>Static abdominal<br>Bridging<br>Forward stretch (modified)<br>Pigeon stretch<br>Squats (easy to increased difficulty, sumo squats in the last trimester)<br>Pelvic tilting and rolling<br>Cat and camel in quadruped position<br>Quadruped—single hand and leg raise, alternate arm and leg raise, arm crossing trunk stretch<br>Child's pose
**Strengthening and resistance exercises**	10 minutes	Various exercises for muscles of upper limb and lower limb with the help of theraband, theratube and dumbell
**Pelvic floor exercises**	10-15 minutes	Kegel (holding and rhythmic/alternate contractions of pelvic muscles) in different positions- supine, sitting, and standing and while doing movements and daily activities-walking, stair climbing, sit to stand, etc<br>Wide squats
**Balance and coordination**	5-10 minutes	Swiss ball pelvic rolls and tilts, trunk stability, forward roll and relaxed belly
**Aerobic exercises**	5 -10 minutes	On the-spot walking, lunges, marching and dancing
**Relaxation**	10 minutes	Rest in supine position with relaxed posture<br>Focusing and feeling of breath<br>"OM" Chanting"
