# Supplementary material for: Effectiveness of different behavioral interventions on gestational weight gain, post-partum weight retention and anthropometric measures in pregnancy: A randomized controlled trial
Source: Health Promot Perspect. 2022 Dec 10;12(3):286–94. doi: 10.34172/hpp.2022.37 (PMC9808912; doi:10.34172/hpp.2022.37)
Supplement: Supplementary file 2 — contains various text messages used in Text Message and Pedometer plus Text Message groups. [file hpp-12-286-s002.pdf]

Rani et al, **Health Promotion Perspectives,** 2022, 12(3), 286-294
doi: 10.34172/hpp.2022.37
https://hpp.tbzmed.ac.ir

Effectiveness of different behavioral interventions on gestational weight gain, post-partum weight retention and anthropometric measures in pregnancy: A randomized controlled trial

Vandana Rani*, Shabnam Joshi

Department of Physiotherapy, Guru Jambheshwar University of Science and Technology, Hisar-125001, Haryana, India

Supplementary file 2. Various text messages used in Text Message and Pedometer plus Text Message groups

General health and well-being	1. Healthy mother healthy baby<br>2. Your body is an asset, keep it fit for healthy baby<br>3. Keep exercising & enjoy exercising for healthy baby<br>4. More active in pregnancy less complications in labour<br>5. Practice healthy life style for healthy baby<br>6. More active lifestyle in pregnancy less labour pain
Physical –activity	1. 30 minutes of regular walking & exercises improves abdominal oxygen of the pregnant women, it promote the brain's development of the child.<br>2. Proper exercises and movement, the amniotic fluid will give a gentle "massage" to the baby's skin, which will also promote the brain development of baby babies.<br>3. Physical activity reduces chances of Leg cramps<br>4. Physical activity reduces and prevents chances of back pain<br>5. Physical activities help and prevent heart burn<br>6. Physical activity helps in easy reduction of post-partum weight<br>7. Physical activity helps in easy delivery<br>8. Physical activity promote caesarean in to normal delivery<br>9. Physical activity helps in good sleep in night<br>10. Physical activity helps in controlling pre gestational diabetics and high blood pressure<br>11. Physical activity enhances and boost up the immune system<br>12. Physical activity helps in prevent constipation
Evidence-based/educational	1. Folic acid is very essential to prevent baby abnormality<br>2. Green vegetables and fruits are rich source of vitamin and iron<br>3. Calcium is essential for baby bone growth so take green vegetables and milk<br>4. Balance diet fulfils calcium, iron and folic acid requirements<br>5. Curd, cereals, dal and egg rich source of protein, so eat them<br>6. For regular hydration drink 10-15 glasses of water<br>7. Never skip medicines & supplements as prescribed by your doctor<br>8. To avoid anaemia or maintain haemoglobin level, eat iron rich food<br>9. Jaggery (gud) and cereals are good source of iron<br>10. Use Jaggery or honey than sugar<br>11. Avoid soft drinks, coffee and fast food<br>12. Use homemade juices or fruits than packed juice<br>13. Paneer, curd & milk are rich source of calcium and iron<br>14. Hard drinks and smoking are injurious to baby health<br>15. Regular check to your doctor
Motivational	1. Good diet decides baby health<br>2. Eat wisely and sleep nicely<br>3. Baby is feeling your each activity, so try to keep yourself happy always<br>4. 9 month are best for every mother so enjoy motherhood
Pregnancy myths	1. Papaya and mango can do miscarriage - Not true<br>2. Excess ghee and oily food eases the delivery - Not true<br>3. Walking and exercises can cause miscarriage - False<br>4. Iron rich diet or tablets can cause dark skin baby - Not true<br>5. Double your diet because you have to eat for two- Not true
